# Adult primary testicular lymphoma: clinical features and survival in a series of patients treated at a high-volume institution in China

**DOI:** 10.1186/s12885-020-6711-0

**Published:** 2020-03-14

**Authors:** Bo Chen, De-Hong Cao, Li Lai, Jian-Bing Guo, Ze-Yu Chen, Yin Huang, Shi Qiu, Tian-Hai Lin, Yue Gou, Na Ma, Lu Yang, Liang-Ren Liu, Qiang Wei

**Affiliations:** 1 Department of Urology, West China Hospital, Sichuan University, No. 37, Guoxue Alley, Chengdu, 610041 Sichuan People’s Republic of China; 2 Institution of Urology, West China Hospital, Sichuan University, No. 37, Guoxue Alley, Chengdu, 610041 Sichuan People’s Republic of China; 3 Department of outpatient, West China Hospital, Sichuan University, Chengdu, 610041 China; 4 West China School of Medicine, Sichuan University, Chengdu, 610041 China

**Keywords:** Primary testicular lymphoma, Diffuse large B-cell lymphoma, Testis

## Abstract

**Background:**

To retrospectively investigate the clinical characteristics, initial treatment, relapse, therapy outcome, and prognosis of Chinese patients with primary testicular lymphoma (PTL) through analysis of the cases of our institute.

**Methods:**

From December 2008 to July 2018, all patients with PTL were included in this study. Kaplan-Meier method was used to estimate PFS and OS. The Cox proportional hazards model was used to compare the survival times for groups of patients differing in terms of clinical and laboratory parameters.

**Results:**

All 28 PTL patients (24 DLBCL, three NK/T lymphomas, and one Burkkit’s lymphoma) with a median age of 65.5 years were included in this study. Six patients were observed recurrence among all the 22 individuals evaluated. Following orchiectomy and systemic chemotherapy, with or without intrathecal prophylaxis, complete response was achieved in 15 (68%) patients. For DLBCL patients, the median progression-free survival (PFS) was 44.63 months (95% CI 17.71–71.56 months), and the median overall survival (OS) was 77.02 months (95% CI, 57.35–96.69 months). For all the DLBCL patients, the 5-year PFS and 5-year OS were 35.4% (95%CI, 14.8–56.0%) and 53.4% (95%CI, 30.1–76.7%). Without further chemotherapy following orchiectomy (HR = 3.4, *P* = 0.03) were associated with inferior PFS of DLBCL patients. Advanced Ann Arbor stage (HR =5.9, *P* = 0.009) and high (international prognostic index, IPI) score: 3–5 (HR =3.9, *P* = 0.04) were correlated with shorter OS of DLBCL patients.

**Conclusion:**

This study confirms that PTL is an aggressive malignant with a poor prognosis. Limited Ann Arbor stage, further chemotherapy following orchiectomy, and low IPI score (less than 2) are correlated with superior survival for DLBCL patients.

## Introduction

Primary testicular lymphoma (PTL) is a rare entity with an annual incidence of 0.26 cases per 100,000 person-years and the most common malignant testicular neoplasms in male over 60 years old, which accounts for about 1–9% in testicular tumors and 1–2% of all non-Hodgkin’s lymphomas [1–3]. The diagnosis of primary testicular lymphoma is usually confirmed through orchiectomy or testis biopsy. Diffuse large B-cell lymphoma (DLBCL) is most common histological subtype of primary testicular lymphoma, comprising 80–90% of all primary lymphoma of testis [1, 4–6]. The most common clinical symptom of PTL is a unilateral painless testicular swelling developing more than weeks to months, even several years. In addition, a minority of patients appear a testicular swelling with sharp pain. Furthermore, bilateral testicular swelling is seen in around 35% of patients [3, 7, 8].

PTL is an extremely aggressive malignant with poor progression-free survival (PFS) and overall survival (OS). PTL performs an inclination to involves the contralateral testis and the central nervous system (CNS), and disseminate to other extranodal sites such as skin, lung, kidney, adrenal, gastrointestinal, and other soft organs [1, 8–10]. A phase 2 study revealed that the 5-year PFS and OS rates were 74 and 85% among limited stage primary testicular DLBCL individuals who received anthracycline-containing chemotherapy in combination with rituximab, prophylactic contralateral scrotal radiotherapy and CNS prophylaxis with intrathecal (IT) chemotherapy [11]. Nevertheless, there are fewer studies providing information for advanced stage PTL patients regarding the survival and outcomes. Recently, several retrospective studies demonstrated improved survival in DLBCL of testis with the addition of rituximab [8, 12]. However, survival improvement has not been observed in other analyses [2]. However, several studies revealed that the addition of rituximab to CHOP (cyclophosphamide, doxorubicin, vincristine, prednisone) chemotherapy results in significant decrease of CNS relapse in PTL [13–15].

The aim of the present work was to retrospectively investigate the clinical characteristics and therapy outcome of Chinese patients diagnosed with PTL through analysis of the cases of our institute.

## Methods

Patients were identified by searching database of West China Hospital of Sichuan University for cases of testicular lymphoma occurring from December 2008 to July 2018. 28 patients with primary PTL were included in this study. The inclusion criteria were signs or symptoms of a testicular mass at presentation, diagnosis of PTL by orchiectomy or needle biopsy, age over 18 years old, and without the history of lymphoma therapy. Moreover, patients who were initially diagnosed with lymphoma outside the testis and who developed a secondary testicular lymphoma were excluded from this study. Therefore, patients with secondary testis involvement were excluded. In addition, all the PTL patients were verified by immunohistochemistry staining. All available clinical files were collected and data concerning age, B symptoms, body mass index (BMI), laterality, tumor size, serum lactate dehydrogenase (LDH), serum β-human chorionic gonadotropin (HCG), serum alpha fetoprotein (AFP), pathology classification, eastern cooperative oncology group (ECOG) score, international prognostic index (IPI), Ann Arbor stage initial treatment, response to treatment, site and time of relapse, and status at final follow-up were recorded. At the same time, because of the limitation of retrospective study, not all variables were available for every individual. Therefore, missing serum LDH was considered to be 0 points when calculating IPI. Then, According to the criteria of our institution: serum LDH ≥220 IU/L, serum β-HCG ≥3.81 mIU/ml,serum AFP ≥8 ng/ml were considered to be elevated.

According to the Ann Arbor criteria, the clinical stage was determined on the basis of medical history, physical examination, blood routine examination, liver and renal function tests, B-ultrasonography, computed tomography, and bone marrow biopsy. StageIindicates that the cancer has mono or bilateral testicular involvement only. StageIIindicates that the tumor with mono or bilateral of the testis involvement is associated with concomitant involvement of loco-regional (peritoneal and/or iliac) lymph nodes. Stage IIIorIV is defined by mono or bilateral testicular involvement with involvement of distant lymph nodes and/or extranodal sites [16]. In addition, B symptoms are defined as a recurrent fever of >38 °C, night sweets, and weight loss >10% within 6 months before diagnosis.

Treatment response of patients is classified according to the definitions recommended by the International Workshop to Standardize Response Criteria for Non-Hodgkin’s Lymphomas [17]. Complete remission (CR) was defined as the disappearance of all detectable clinical and radiographic evidence of disease and disappearance of all disease-related symptoms present before therapy. Partial remission (PR) was defined as a ≥ 50% reduction in tumor bulk. Stable disease (SD) was defined as less than a partial remission but not progressive disease. Progressive disease (PD) was defined as a ≥ 50% increase in the sum product of the greatest diameters of any previously identified abnormal tumor bulk or the appearance of any new signs of disease during or at the end of therapy. Overall survival (OS) was measured from the time of diagnosis to the time of death from any cause or of last follow-up. Progression-free survival (PFS) was measured from the time of diagnosis to the time of the disease progression, the disease relapse, the latest check-up, or death from lymphoma. Moreover, Patients treated only by testis biopsy, were considered as being in PD, who did not receive any treatment after testis biopsy. Then, patients treated only by surgery were considered as being in CR if no signs of disease were noted after orchiectomy.

Kaplan-Meier method was used to estimate PFS and OS. Differences between curves were analyzed by using the log-rank test. The chi-square test was used to detect statistically significant differences for categorical variables. The Cox proportional hazards model was used to compare the survival times for groups of patients differing in terms of clinical and laboratory parameters. Analyses were carried out using SPSS 21.0 software package (Chicago, IL, USA). Reporting of all the analysis is agreement with guidelines for reporting of statistics in European Urology [18].

## Results

### Clinical characteristics

A summary of the main clinical characteristics of all patients is presented in Table 1. Twenty-eight male patients diagnosed with PTL with a median age of 65.5 years (IQR 56.5–72.8 years) met the eligibility criteria for our study. The most common initial symptom of patients was unilateral or bilateral swelling of testis, accompanied by pain in few cases. Then, interestingly, B symptoms were absent in 25 (89%) patients; and three (11%) patients were unknown. The median tumor size is 5.0 cm (IQR 4.1–7.1 cm). Serum LDH is elevated in 14 (50%) patients, and unknown in three (11%) patients. In addition, 8 (29%) patients, 16 (57%) patients and 4 (14%) patients had low (0–1 risk factors), intermediate (2–3 risk factors) or a high (4–5 risk factors) IPI score, respectively. The Ann Arbor clinical stage was as follows: stage Iin 14 (50%) patients, stage II in five (18%) patients, stage III in three (11%) patients, stage IV in 4 (14%) patients, and stage unknown in two (7%) patients. The majority of advanced stage disease had additional extranodal sites including prostate, urinary bladder, kidney, adrenal gland, lung, heart, and other soft tissues.

**Table 1:** Clinical characteristics

characteristics	No. of patients	%
Age (years)		
Median	65.5	
IQR	56.5–72.8	
B symptoms		
Absent	25	89
Present	0	0
Unknown	3	11
BMI		
Median	21.7	
IQR	20.0–25.6	
Laterality		
Left	11	39
Right	13	47
Bilateral	4	14
Tumor size (cm)		
Median	5.0	
IQR	4.1–7.1	
Serum LDH		
Normal	11	39
Elevated	14	50
Unknown	3	11
Serum β-HCG (mIU/ml)		
Median	0.4	
IQR	0.08–1.2	
Serum AFP (IU/L)		
Median	2.53	
IQR	1.66–3.42	
Pathology classification		
DLBCL	24	85
NK/T lymphoma	3	11
Burkitt’s lymphoma	1	4
ECOG score		
0–1	12	43
≥ 2	16	57
IPI		
0–1	8	29
2–3	16	57
4–5	4	14
Stage		
I	14	50.
II	5	18
III	3	11
IV	4	14
Uknown	2	7

Table 2 Note:*IQR* Interquartile range, *BMI* Body mass index, *LDH* Lactate dehydrogenase, *HCG* Human chorionic gonadotropin, *AFP* Alpha fetoprotein, *NK/T* Natural killer/T, *ECOG* Eastern cooperative oncology group, *IPI* International prognostic index. Missing serum LDH and unknown stage were considered to be 0 points when calculating IPI.

### Pathological characteristics

Immunochemistry staining was performed in all 28 patients. 24 out of 28 patients (85%), three cases (11%), sole one (4%) were confirmed DLBCL, NK/T lymphoma and Burkitt’s lymphoma, respectively. Table 2 summarizes immunohistochemistry characteristics patients with PTL. Firstly, All the DLBCL were CD20+. Interestingly, both CD10 and CD3 were negative in 23 patients (96%), and positive in one patient (4%). On the contrary, 23 cases (96%) out of 24 DLBCL were Mum-1+ and one tumor (4%) was Mum-1-. In addition, it is similar to Mum-1 that bcl-6 was weakly positive in two patients. Median of Ki-67 expression in DLBCL and PTL is 80% (IQR 61.3–88.8% in DLBCL).

**Table 2:** Results of immunohistochemistry staining of all patients with primary testicular lymphoma

Patient no	Classification	*GCB/non-GCB*	*CD20*	CD3	CD10	Mum-1	bcl-6	Ki-67
1	DLBCL	na	+	–	–	+	+	40%
2	DLBCL	non-GCB	+	–	–	+	+	95%
3	DLBCL	GCB	+	–	+	–	+	80–90%
4	DLBCL	non-GCB	+	–	–	+	–	80–90%
5	DLBCL	na	+	–	–	+	+	75%
6	DLBCL	non-GCB	+	–	–	+	+	90%
7	DLBCL	non-GCB	+	–	–	+	+	85%
8	DLBCL	na	+	–	–	+	+	60%
9	DLBCL	non-GCB	+	+	–	+	+	40%
10	DLBCL	non-GCB	+	–	–	+	+	50%
11	DLBCL	non-GCB	+	–	–	+	+	50–60%
12	DLBCL	na	+	–	–	+	na	50%
13	DLBCL	non-GCB	+	–	–	+	–	80%
14	DLBCL	non-GCB	+	–	–	+	+	70%
15	DLBCL	na	+	–	–	+	+	90%
16	DLBCL	non-GCB	+	–	–	+	+	60–70%
17	DLBCL	non-GCB	+	–	–	+	–	80%
18	DLBCL	non-GCB	+	–	–	+	+	80%
19	DLBCL	non-GCB	+	–	–	+	+	90%
20	DLBCL	non-GCB	+	–	–	+	+	90%
21	DLBCL	non-GCB	+	–	–	+	–	50%
22	DLBCL	non-GCB	+	–	–	+	+	80%
23	DLBCL	non-GCB	+	–	–	+	+	85%
24	DLBCL	non-GCB	+	–	–	+	+	80%
25	NK/T lymphoma		–	+	–	–	–	80% 95% 80% 99%
26	NK/T lymphoma		–	–	–	–	–	95%
27	NK/T lymphoma		–	+	na	na	na	80%
28	Burkitt’s lymphoma		+	–	+	na	+	99%

**Note:***NK/T* Natural killer/T, *na* Not available, *GCB/non-GCB* Germinal center B/non-germinal B

The immunohistochemistry of NK/T lymphoma and Buekkit’s lymphoma was not presented in Table 2 because of few patients. Moreover, other markers such as granzyme B, CD30, PLAP, CD79a were not performed in Table 2 because these markers were not reported by pathologists.

### Initial treatment

An overview of the main clinical data, treatment modalities, and outcomes is presented in Table 3. Twenty-six patients (93%) underwent orchiectomy, including four patients with bilateral orchiectomy and 22 patients with unilateral orchiectomy, as first therapeutic and diagnostic intervention. Two patients (7%) received testis biopsy to confirm diagnosis. In our study, there are eight patients without further treatment.

**Table 3:** Patients treatment and outcomes

Patient no	*Stage*	*Surgery*	Further therapy	Response	Time to relapse (months)	Site of relapse	Overall survival (months)
1	I	Uni	CHOP+RT + IT	CR	38.77	skin, CNS	40.77
2	II	Bil	R-CHOP+CHOP	CR			116.47+
3	IV	Uni	No	PD			6.07
4	I	Bil	unknown	unknown	unknown	unknown	112.1+
5	unknown	Uni	unknown	unknown	unknown	unknown	89.97+
6	II	Uni	R-CHOP+CHOP	PR			3.4+
7	II	Uni	R-CHOP	CR			79.27
8	I	Uni	R-CHOP+RT + IT	CR	71.2	maxillary sinus	78.37+
9	I	Uni	unknown	unknown	unknown	unknown	77.53+
10	I	Uni	R-CHOP+IT	CR	54.23	CNS	76.63+
11	III	Uni	R-CHOP+IT	CR			71.8+
12	I	Uni	R-CHOP+RT + IT	CR			69.53+
13	III	Uni	R-CHOP+RT + IT	SD			28
14	I	Uni	No	CR			44.63+
15	IV	Uni	R-CHOP+IT	PD			3.1
16	I	Uni	No	CR			34.93+
17	I	Uni	unknown	unknown	unknown	unknown	34.17+
18	II	Uni	R-CHOP+RT + IT	CR			33.47+
19	I	Uni	No	CR	12.77	right neck	21.97
20	I	Uni	No	CR	8.6	soft tissue	16.2
21	I	Uni	RT	CR	2.6	right testis	53.63+
22	unknown	Biopsy	unknown	unknown	unknown	unknown	28.9+
23	I	Uni	R-CHOP	CR			11.8+
24	I	Uni	R-CHOP	CR			4.63+
25	II	Uni	unknown	unknown	unknown	unknown	93.23+
26	IV	Bil	No	PD			22.57
27	III	Bil	No	PD			35.27
28	IV	Biopsy	No	PD			12.17

Table 3 note: *Uni* Unilateral; Bil, Bilateral, *CHOP* (cyclophosphamide, doxorubicin, vincristine, prednisone), *R-CHOP (rituximab* Cyclophosphamide, doxorubicin, vincristine, prednisone), *RT* Radiotherapy, *IT* Intrathecal, *CR* Complete remission, *SD* Stable disease, *PD* Progressive disease, *PR* Partial remission, *CNS* Central nervous system.

Prophylactic radiotherapy (RT) to the contralateral testis was given to five patients, and one patient had additional radiation to the involved lymph node areas.

In total, twelve patients (43%) out of 28 patients were administrated with systemic chemotherapy after orchiectomy. One patient (4%) received CHOP (cyclophosphamide, doxorubicin, vincristine, prednisone) chemotherapy, and nine patients (32%) received R-CHOP (rituximab, cyclophosphamide, doxorubicin, vincristine and prednisone), and two patients (7%) received R-CHOP plus CHOP chemotherapy. Besides systemic chemotherapy, in eight patients (29%), intrathecal prophylaxis (IT) was delivered with 15 mg methotrexate (MTX) plus 5 mg dexamethasone or 50 mg cytarabine plus 5 mg dexamethasone or 15 mg MTX plus 30 mg cytarabine plus 5 mg dexamethasone.

Therefore, only five patients received combined modality therapy of systemic chemotherapy, RT, and IT.

### Outcome

Above all, at a median follow-up time of 74.29 months (IQR 34.36–84.81 months), six patients (21%) were lost to follow-up. Thus, twenty-two patients out of 28 patients were evaluable for initial therapy response. CR was achieved in 15 (68%) patients, including 14 limited stage patients (stageIor II) and only one advanced stage patient (III or IV). In addition, PR was observed in one (5%) stage II patient and SD was recognized in one (5%) stage III patient. Furthermore, PD was detected in five patients (23%). It’s worth noting that all patients with disease progression are advanced stage and four patients out of five patients didn’t receive further treatment after orchiectomy.

**Fig. 1:**
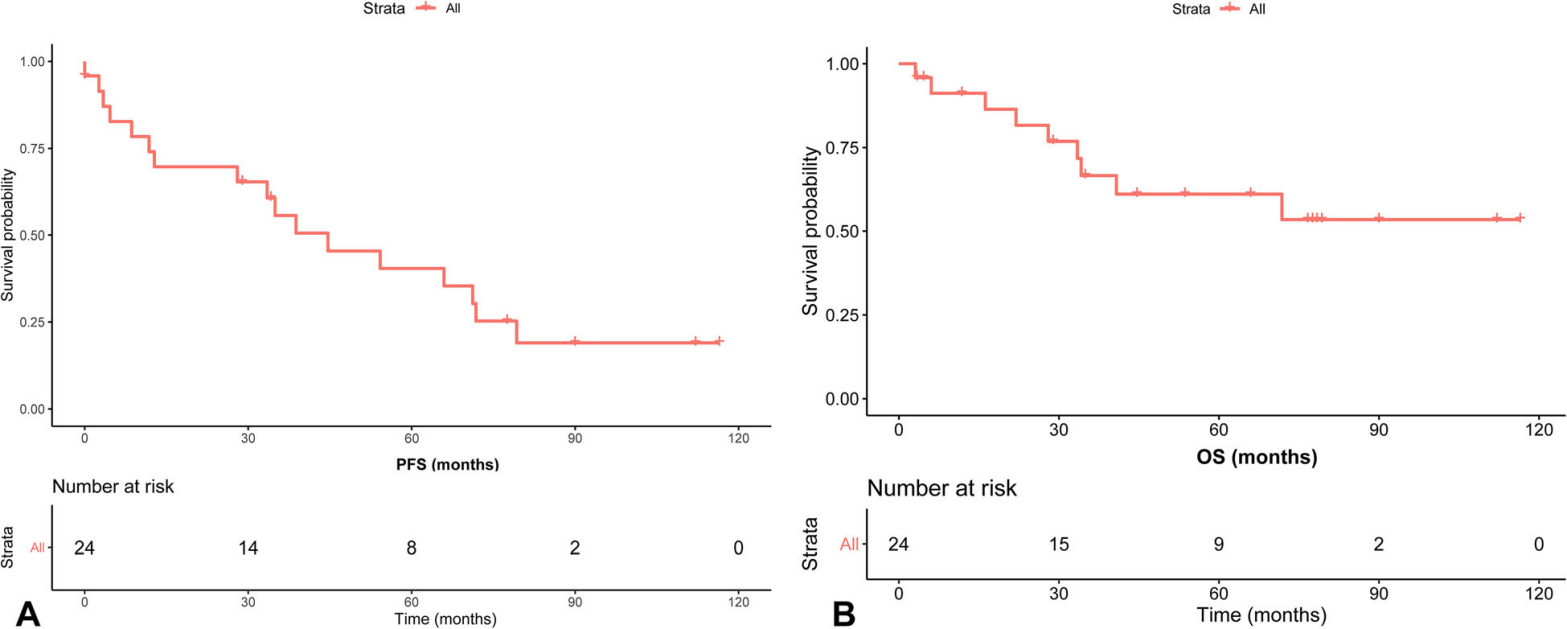
Kaplan-Meier survival curves showing (**a**) progression-free survival (PFS) and (**b**) overall survival (OS) of DLBCL patients

The Kaplan-Meier estimated median PFS of all the DLBCL patients was 44.63 months (95% CI 17.71–71.56 months) as shown in Fig. 1a, and the median OS was 77.02 months (95% CI 57.35–96.69 months) as shown in Fig. 1b. For all the DLBCL patients, the 5-year PFS and 5-year OS were 35.4% (95%CI, 14.8–56.0%) and 53.4% (95%CI, 30.1–76.7%).

### Relapse

In the 22 patients who we could evaluate, 6 (27%) had relapsed, with a median time to relapse of 33.25 months (range: 2.40–70.24 months). The extranodal sites were as follows: CNS, contralateral testis, soft tissue, right neck, maxillary sinus. In the eight patients treated with intrathecal chemotherapy, two (25%) patients relapsed in the CNS. Furthermore, two patients with CNS relapse received IT, which was administered concurrently with systemic chemotherapy. The IT regimens of the first patient with CNS relapse were 15 mg MTX plus 5 mg dexamethasone for one time and 50 mg cytarabine plus 5 mg dexamethasone for twice. Then, the IT regimen of another patient with CNS relapse was 15 mg MTX plus 30 mg cytarabine plus 5 mg dexamethasone for three times. While in those not treated with intrathecal chemotherapy, nobody had a relapse of CNS. Furthermore, we recognized that one patient, who was administrated with prophylactic radiotherapy only without chemotherapy, had relapsed in the contralateral testis. In addition, two patients with relapse didn’t receive any therapy after orchiectomy. Then, one patient with relapse was treated with systemic chemotherapy, RT, and IT.

In our series, among the 6 individuals with relapsed disease, five patients received the second line chemotherapies. Two cases were administrated with R-MA (rituximab, methotrexate and cytarabine), and three patients received R-ICE (rituximab, ifosfamide, carboplatin and etoposide), and a single patient did not receive any further therapy at disease relapse. Moreover, CR was achieved in three patients (one received R-MA and two received R-ICE). PD was observed in three cases (one received R-MA, one received R-ICE and one without further therapy). Regrettably, none of the patients underwent stem cell transplantation (SCT) in our series.

Finally, in patients with known relapse, patients who had received R-CHOP based treatment relapsed at 38.77, 71.2, 54.23 months, respectively. In contrast, other patients relapsed at 12.77, 8.6, 2.6 months respectively. This marked difference demonstrated that standard chemotherapy is strongly encouraged even though it may not be curative for most patients.

### Prognostic factors for DLBCL

**Fig. 2:**
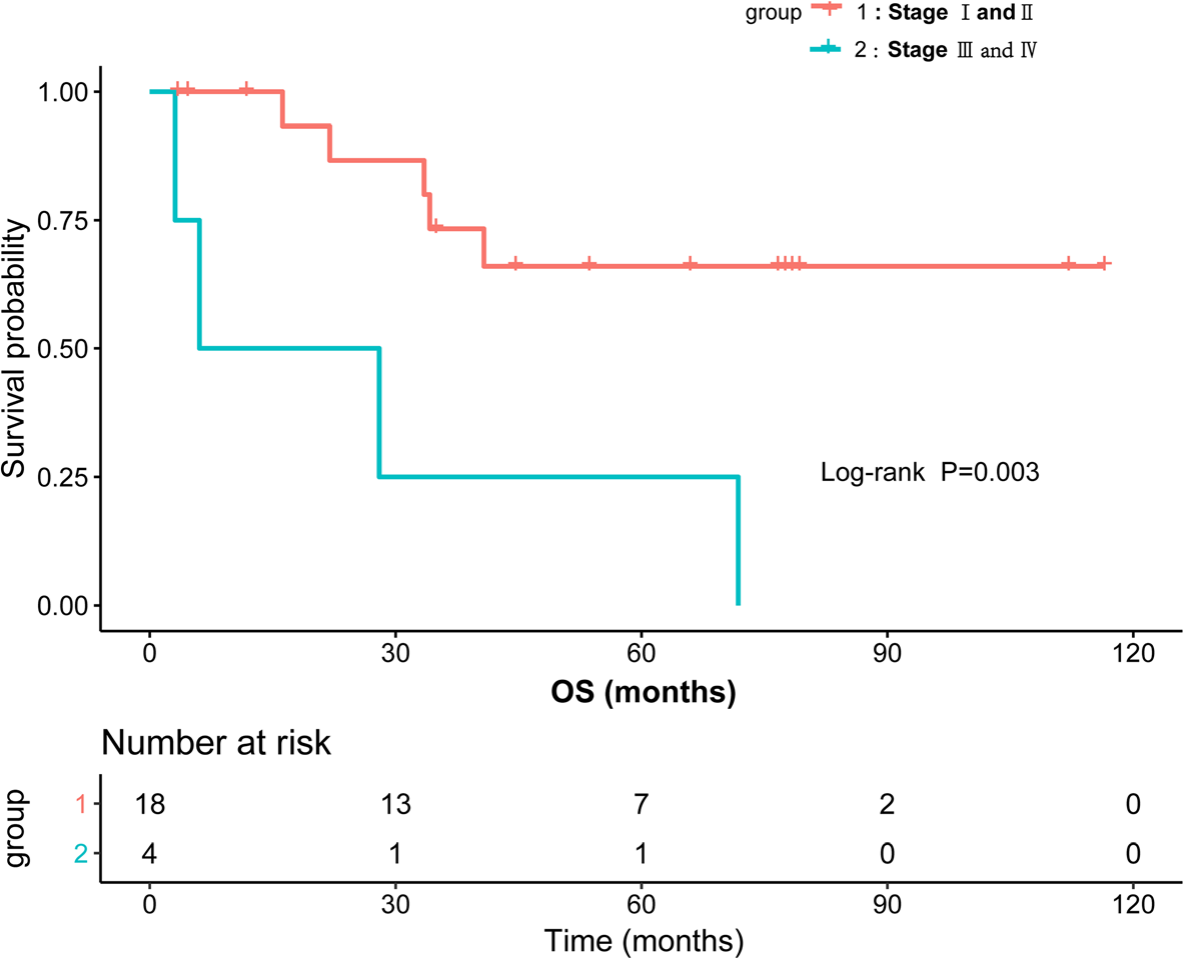
Kaplan-Meier survival curves showing overall survival (OS) of DLBCL patients by Ann Arbor stage

**Fig. 3:**
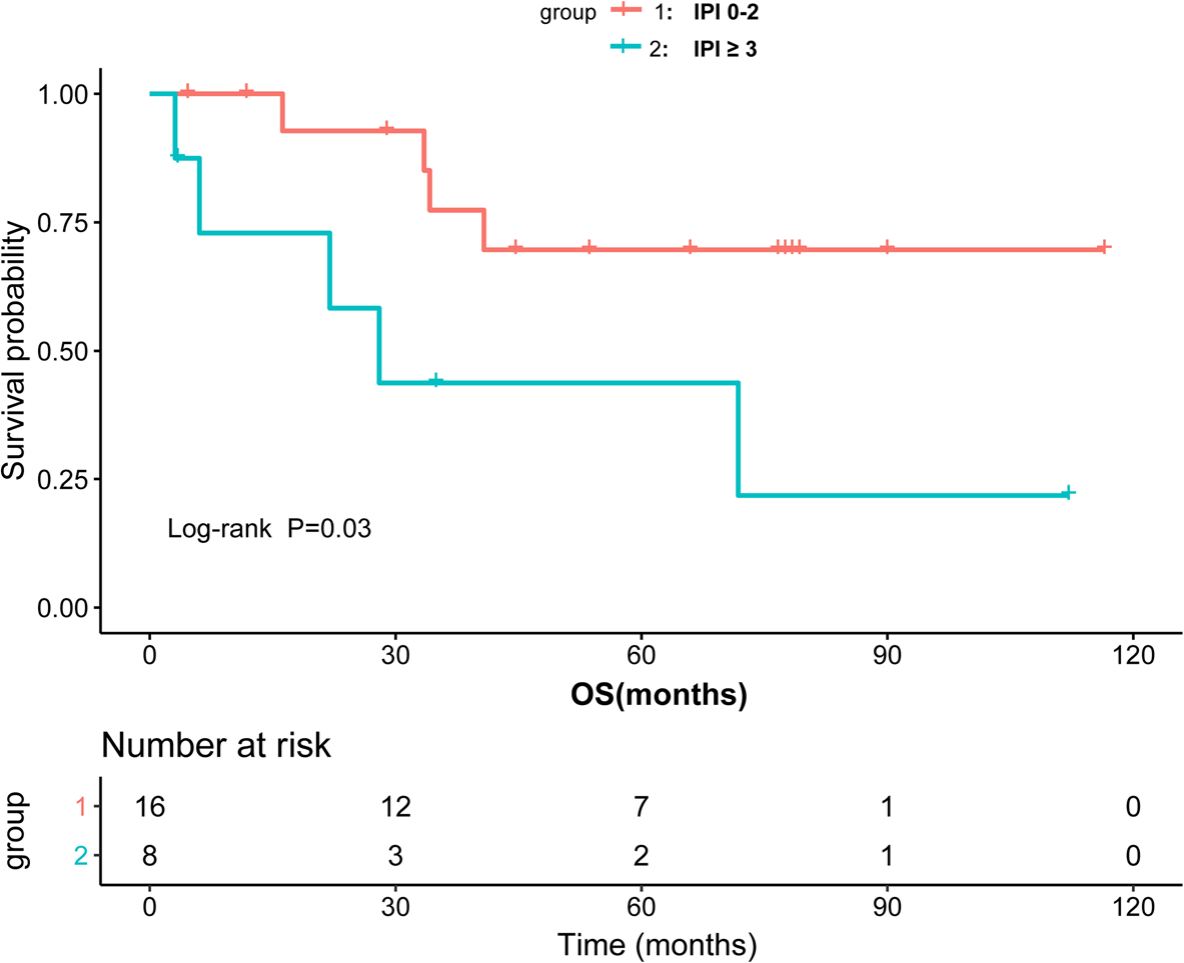
Kaplan-Meier survival curves showing overall survival (OS) of DLBCL patients by international prognostic index (IPI) score

Ann Arbor stage and IPI score were associated with OS. The 5-year OS was 66.0% in patients with stageIor II versus 25.0% in patients with stage III or IV (Log-rank *P* = 0.0009). The median OS time of patients with stage III or IV was 27.24 months (95%CI, 2.84–51.64 months), as shown in Fig. 2. In our study, IPI score was significantly associated with patients OS. The 5-year OS was 69.6% in patients with IPI score 0–2 versus 21.9% in patients with high IPI score 3–5 (Log-rank *P* = 0.04). The median OS time of patients with high IPI score 3–5 was 28.00 months (95%CI, 12.78–43.23 months), as shown in Fig. 3.

A Cox proportional hazards model including Ann Abor stage, serum LDH, IPI score, ECOG score, location, age, further chemotherapy, further radiation, intrathecal prophylaxis and tumor size was constructed. In the univariable cox hazard ratio model, analysis of factors influencing PFS and OS of DLBCL patients is summarized in Table 4. Without first line chemotherapy following orchiectomy (HR = 3.4, *P* = 0.03) was associated with a significantly shorter PFS. Factors associated with a significantly shorter OS included advanced Ann Arbor stage disease (HR =5.9, *P* = 0.009), high IPI score: 3–5 (HR =3.9, *P* = 0.04).

**Table 4:** Univariable cox proportional hazard model for independent effects of Ann Abor stage, serum LDH, IPI score, ECOG score, location, age, further chemotherapy, further radiation, intrathecal prophylaxis, and tumor size on progression-free survival (PFS) and overall survival (OS)

Variable	PFS		OS	
	HR	*P*-value	HR	*P*-value
Advanced stage (III or IV) vs. limited stage (I or II)	1.6(95%CI, 0.5–5.9)	0.5	5.9(95%CI,1.6–25.0)	**0.009**
Elevated serum LDH vs. normal serum LDH	1.0(95%CI,0.4–2.7)	0.9	1.6(95%CI,0.4–6.3)	0.5
IPI score: 3–5 vs. 0–2	1.6(95%CI,0.2–2.7)	0.4	3.9(95%CI,1.1–16.7)	**0.04**
ECOG score over 1 vs. not more than 1	1.0(95%CI,0.4–2.7)	0.9	1.7(95%CI,0.4–6.7)	0.5
Location: unilateral vs bilateral	9.0(95%CI,0.1–36.8)	0.2	8.1(95%CI,0.06–42.0)	0.5
Location: left vs. others	1.2(95%CI,0.5–3.2)	0.7	0.8(95%CI,0.2–3.2)	0.7
Location: right vs. others	1.8(95%CI, 0.7–4.7)	0.3	2.2(95%CI, 0.6–8.8)	0.3
Age ≥ 60 vs. age < 60	0.9(95%CI,0.3–2.6)	0.8	1.2(95%CI,0.3–5.8)	0.8
Further chemotherapy: NO vs. YES	3.4(95%CI,1.1–10.9)	**0.03**	1.8(95%CI,0.4–8.0)	0.5
Further radiation: NO vs. YES	0.8(95%CI,0.3–2.3)	0.7	1.1(95%CI,0.3–4.7)	0.9
Intrathecal prophylaxis: NO vs. YES	1.0(95%CI,0.4–2.7)	0.9	0.6(95%CI,0.1–2.6)	0.5
Tumor size (cm):≥5 vs. <5	0.7(95%CI, 0.3–2.1)	0.6	1.5(95%CI, 0.4–5.5)	0.6

Table 4 note: *LDH* Lactate dehydrogenase, *IPI* International prognostic index, *ECOG* Eastern cooperative oncology group.

Due to the fact that “further chemotherapy following orchiectomy” was the unique variable associated with PFS significantly in univariable cox hazard ratio analysis, we didn’t perform a multivariable model in terms of PFS. With regard to OS, then, we create a multivariable model including the following variables: stage and IPI score. We find that high IPI score: 3–5 was associated with a marked inferior OS (HR = 5.3, 95%CI 1.4–19.7, *P* = 0.01).

## Discussion

Our study confirms that primary testicular lymphoma (PTL) is a rare malignant with poor prognosis. The median age of presentation in our study (65.5 years) was on par with other studies [1, 3, 19], which reported that PTL is most common in male over 60 years. It is worth noting that the OS of patients with testicular lymphoma had gradually improved over the past decades. In the early years, the treatment of PTL included orchiectomy, followed chemotherapy and radiation. Until to 1995, a combined modality therapy was recommended to PTL, which consists of orchiectomy, systemic chemotherapy, scrotal radiotherapy, and prophylaxis intrathecal chemotherapy [20]. It is extensive agreement that orchiectomy is the main diagnostic approach and first therapy in PTL. Therefore, survival improvement of PTL maybe was contributed to doxorubicin based chemotherapy (CHOP, R-CHOP), scrotal radiotherapy, and prophylaxis intrathecal. Moreover, it was also shown to be true in our study that a combined modality therapy could promote PTL patients survival.

Patients with PTL have a 10–15% increase in survival because of the incorporation of rituximab into standard therapy with CHOP with minimal added toxicity. The benefit of rituximab for DLBCL has been provided in patients with limited stage disease [21, 22] and advanced stage disease [23–25]. In 2017, Robert Kridel et al. [10] demonstrated that rituximab was associated with significantly improved PFS, OS and cumulative incidence of testicular lymphoma progression, which is agreement with observation results in nodal DLBCL [21, 23, 25]. In our study, we didn’t compare R-CHOP group with CHOP group because of small size sample and only two patients receiving pure CHOP. However, we found that patients received further chemotherapy or not was significantly associated with outcome.

The role of IT chemotherapy as CNS prophylaxis is still a matter of debate. Zouhair et al. [26] noted that CNS relapse fraction in patients received IT prophylaxis is same to those who didn’t receive IT prophylaxis. Furthermore, Zucca et al. [1] carried out a study which demonstrated improved PFS among a small subset of patients administrated with IT prophylaxis but not a statistically significant reduction in the CNS relapse rate. Then, in the eight patients treated with intrathecal chemotherapy, two (25%) patients relapsed in the CNS. While in those not treated with intrathecal chemotherapy, nobody had a relapse of CNS. Thus, CNS prophylaxis with IT in our institute was failure based on this experience. Nevertheless, the failure experience does not entirely suggest IT is not useful, as the intensity of IT may not be adequate in these patients. Therefore, further studies are needed to explore adequate intensity of CNS prophylaxis IT. Furthermore, a statistically significant improvement was not recognized in patients treated with prophylactic intrathecal methotrexate or cytarabine in our study. One of potential reasons is that our study sample was too small to acquire statistically significant result. Another reason contributes to this result might be that intrathecal prophylaxis distinctly could not improve PFS and OS. In addition, maybe the third one reason was that only advanced Ann Arbor stage patients treated with IT prophylaxis rather than limited Ann Arbor stage patients.

Of interest, it is worth noting that one patient occurred relapse of contralateral testis within 2 months who only received radiation after orchiectomy. Furthermore, a significantly decreased contralateral testis relapse rate in patients with prophylaxis radiotherapy was not indicated in our study maybe because of small sample. Nevertheless, the benefit of prophylactic radiation to reduce the risk of contralateral testis relapse has been confirmedly demonstrated in some studies [1, 27]. In addition, we have to emphasis that a majority of patients with PTL were over 60 years again. Therefore, it is not greatly necessary to preserve testicular function so that prophylactic radiation to the contralateral testis should be taken into physician consideration.

A tendency of PTL spreading to extranodal site, including CNS, contralateral testis, lung, kidney, adrenal gland and soft tissues, has been reported in a large number of studies [6, 8, 10, 28, 29]. In our study, the extranodal sites of PTL dissemination, including CNS, contralateral testis, kidney, adrenal gland, maxillary sinus, and soft tissue, which is in universal agreement with other studies. Nevertheless, the reason for this preferential involvement in other extranodal sites remains unknown. Potential explanation including, (1) the efficacy of chemotherapy will be decreased in CNS and contralateral testis due to the blood brain barrier and blood-testis barrier [1]; (2) lacking of expression of integrin and adhesion molecules in PTL resulting in poor adhesion of tumor cells to the extracellular matrix [30, 31]; and (3) CD44 variant plays significant roles in lymphoma dissemination [32].

It has been reported that better PFS and OS were associated with good performance status, limited stage, low IPI score, absence of B symptoms, normal serum LDH, and β_2_-microglobulin, absence of additional extranodal sites involvement, and right testis involvement [1, 2, 8, 28, 29]. Then, we carried out univariate analysis in our study and found that the prognostic factors associated with a poor outcome included advanced Ann Arbor stage, without further chemotherapy after orchiectomy, and high IPI score, which is universal consistent with other reports [6, 8, 10, 28, 29, 33]. Owing to extremely rare incidence of NK/T cell lymphoma and Burkitt’s lymphoma in testis, to the best of our knowledge, a majority of study of them are limited to small case series only or case reports [34–39]. Nevertheless, Besides DLBCL, NK/T cell lymphoma and Burkitt’s lymphoma were taken into our study too.

A meta-analysis to investigate high dose chemotherapy plus autologous SCT in the first line therapy of non-Hodgkin’s lymphoma patients manifested that OS showed no significant difference between high dose chemotherapy plus autologous SCT and conventional chemotherapy (HR1.0, 95%CI 0.9–1.2, *P* = 0.6) as well as event free survival (HR0.9, 95% CI 0.8 to 1.1, *P* = 0.3), and CR rates were significantly higher in the group receiving high dose chemotherapy plus autologous SCT than conventional chemotherapy [40]. Nevertheless, another study demonstrated that high dose chemotherapy plus autologous SCT significantly increase event free and OS compared with conventional chemotherapy in relapsed non-Hodgkin’s lymphoma patients [41]. Thus, high dose chemotherapy and autologous SCT are considered for relapsed PTL in view of PTL as a rare disease with poor prognosis.

We recognize that this retrospective study has potential shortcomings. Firstly, due to the retrospective nature, data of six patients are missing in our study, including further treatment, response to therapy, time to relapse. The rate of lost to follow-up (21%) in our study closes to the rate of lost to follow-up accepted by academia (20%). Therefore, the results and conclusion of our study is credible. Secondly, another limitation of our study is that the Kaplan-Meier and Cox proportional hazards model were not performed to analysis the survival and prognostic factors of NK/T lymphoma and Buekkit’s lymphoma cases in view of few cases. Therefore, multi-centers, large sample and prospective studies are needed to investigate the survival and prognostic factors of NK/T lymphoma and Buekkit’s lymphoma individuals. However, serum AFP and β-HCG were summarized in our study, which were not reported in other studies about PTL. Moreover, we found that neither serum AFP nor β-HCG was elevated in any patient of our study. Therefore, what do we want to demonstrate is that PTL should be taken into physician’s consideration when both serum AFP and serum β-HCG are normal in patients with testis swelling.

## Conclusion

This study confirms that PTL is an aggressive malignant with a poor prognosis. Limited Ann Arbor stage, further chemotherapy following orchiectomy, and low IPI score (less than 2) are correlated with superior survival for DLBCL patients. Thus, systemic treatments, including orchiectomy, chemotherapy, radiotherapy, and intrathecal prophylaxis, are necessary for all the patients with PTL.

## Data Availability

All data generated or analyzed during the present study are included in this published article. The authors declare that materials described in the manuscript, including all relevant raw data, will be freely available to any scientist wishing to use them for non-commercial purposes, without breaching participant confidentiality.

## Abbreviations

PTL: Primary testicular lymphoma
DLBCL: Diffuse large B-cell lymphoma
GCB: Germinal center B
NK/T: Natural killer/T
PFS: Progression free survival
OS: Overall survival
CNS: Central nervous system
IELSG: Extranodal Lymphoma Study Group
IT: Intrathecal
RT: Radiotherapy
CHOP: Cyclophosphamide, doxorubicin, vincristine and prednisone
R-CHOP: Rituximab, cyclophosphamide, doxorubicin, vincristine and prednisone
R-MA: Rituximab, methotrexate and cytarabine
R-ICE: Rituximab, ifosfamide, carboplatin and etoposide
BMI: Body mass index
LDH: lactate dehydrogenase
HCG: Human chorionic gonadotropin
AFP: Alpha fetoprotein
ECOG: Eastern cooperative oncology group
IPI: International prognostic index
CR: Complete remission
PR: Partial remission
SD: Stable disease
PD: Progressive disease
MTX: Methotrexate
CI: Confidence interval
HR: Hazard ratio
SCT: Stem cell transplantation
